# Comprehensive analysis of prognostic value, immune implication and biological function of CPNE1 in clear cell renal cell carcinoma

**DOI:** 10.3389/fcell.2023.1157269

**Published:** 2023-04-03

**Authors:** Haiting Zhou, Yi He, Yongbiao Huang, Rui Li, Hao Zhang, Xiaohui Xia, Huihua Xiong

**Affiliations:** ^1^ Department of Oncology, Tongji Hospital, Huazhong University of Science and Technology, Wuhan, China; ^2^ Department of Orthopedics, Tongji Hospital, Huazhong University of Science and Technology, Wuhan, China

**Keywords:** CPNE1, prognosis, immune infiltration, renal cell carcinoma, EGFR/STAT3

## Abstract

**Background:** Elevated expression of Copine-1 (CPNE1) has been proved in various cancers; however, the underlying mechanisms by which it affects clear cell renal cell carcinoma (ccRCC) are unclear.

**Methods:** In this study, we applied multiple bioinformatic databases to analyze the expression and clinical significance of CPNE1 in ccRCC. Co-expression analysis and functional enrichment analysis were investigated by LinkedOmics, cBioPortal and Metascape. The relationships between CPNE1 and tumor immunology were explored using ESTIMATE and CIBERSORT method. *In vitro* experiments, CCK-8, wound healing, transwell assays and western blotting were conducted to investigate the effects of gain- or loss-of-function of CPNE1 in ccRCC cells.

**Results:** The expression of CPNE1 was notably elevated in ccRCC tissues and cells, and significantly correlated with grade, invasion range, stage and distant metastasis. Kaplan–Meier and Cox regression analysis displayed that CPNE1 expression was an independent prognostic factor for ccRCC patients. Functional enrichment analysis revealed that CPNE1 and its co-expressed genes mainly regulated cancer-related and immune-related pathways. Immune correlation analysis showed that CPNE1 expression was significantly related to immune and estimate scores. CPNE1 expression was positively related to higher infiltrations of immune cells, such as CD8^+^ T cells, plasma cells and regulatory T cells, exhibited lower infiltrations of neutrophils. Meanwhile, elevated expression of CPNE1 was characterized by high immune infiltration levels, increased expression levels of CD8^+^ T cell exhaustion markers (CTLA4, PDCD1 and LAG3) and worse response to immunotherapy. *In vitro* functional studies demonstrated that CPNE1 promoted proliferation, migration and invasion of ccRCC cells through EGFR/STAT3 pathway.

**Conclusion:** CPNE1 is a reliable clinical predictor for the prognosis of ccRCC and promotes proliferation and migration by activating EGFR/STAT3 signaling. Moreover, CPNE1 significantly correlates with immune infiltration in ccRCC.

## 1 Introduction

Renal cell carcinoma (RCC) is one of the most frequently observed malignancies in the urinary system. Its incidence is about 3%, and the 5-year mortality rate is as high as 40% in adult malignancies (Siegel et al., 2022). In recent years, with the continuous improvement and popularization of diagnosis and treatment technology, the incidence of RCC has an obvious upward trend. Clear cell renal cell carcinoma (ccRCC) is the most common pathological type of RCC, accounting for about 75%–80% (Paner et al., 2018). Currently available kinase inhibitors and immune checkpoint inhibitors greatly improved the therapeutic effect (Barata and Rini, 2017; Zhang and George, 2021). However, there are still some challenges to be solved, such as, not all patients with ccRCC receive clinical benefits from these therapies, the development of drug resistance, loss of efficacy of a particular drug (Rini and Atkins, 2009; Posadas et al., 2017). Therefore, it is imperative to further study the mechanisms of ccRCC progression in order to obtain more effective anti-tumor therapy.

Copines are a family of ubiquitously distributed and highly conserved Ca2^+^-dependent phospholipid-binding proteins during evolution, and share common structural features: two C2 domains at the N-terminus, which act as Ca2^+^-dependent and phospholipid-binding properties and may be involved in cell signal transduction and cell membrane transport; a Von Willebrand factor A (VWA) domain at the C-terminus, which can mediate the interaction between extracellular matrix proteins and may be served as a protein-binding domain (Creutz et al., 1998; Tomsig and Creutz, 2000; Tomsig et al., 2003; Park et al., 2014). In mammals, nine members of copines family have been identified.

Copine-1 (CPNE1) is located on chromosome 20q11.21 region coding 537-amino acid protein (Yang et al., 2008). CPNE1 is involved in multiple cellular biological processes such as proliferation, apoptosis, autophagy, inflammation, exocytosis, gene transcription, and cytoskeletal organization (Ilacqua et al., 2018). Multiple studies have conclusively proven that CPNE1 was upregulated in malignancies and closely associated with the occurrence and development of cancer. In breast cancer, CPNE1 predicts adverse prognosis and facilitates tumorigenesis and radio-resistance through the AKT pathway (Shao et al., 2020). In lung cancer, CPNE1 acts as a target of miR-335-5p, and the inhibition of CPNE1 could enhance the clinical efficacy of EGFR-tyrosine kinase inhibitors (Tang et al., 2018a). In colorectal cancer, CPNE1 increased aerobic glycolysis *via* regulating AKT-GLUT1/HK2 pathway, and contributed to chemoresistance (Wang et al., 2021). In osteosarcoma, CPNE1 significantly promoted cell proliferation, colony formation, invasion and metastasis (Jiang et al., 2018a). In prostate cancer, CPNE1 proved to be a significant prognostic biomarker for evaluating recurrence-free survival, and was positively correlated with TRAF2 expression (Liang et al., 2017). In liver cancer, knockdown of CPNE1 inhibited proliferation, migration and invasion *via* the AKT/P53 signaling (Su et al., 2022). A recent study indicated that CPNE1 might serve as an independent prognostic biomarker for ccRCC by bioinformatics analysis and immunohistochemical staining (Talaat et al., 2022). However, to date, the biological function, molecular mechanisms and immune implication of CPNE1 in ccRCC remains unknown.

In the present study, we performed a series of bioinformatic analyses to investigate expression level and the potential biological functions of CPNE1 in ccRCC, and explored the relationship between CPNE1 and immunology in ccRCC. In addition, gain- or loss-of-function of CPNE1 *in vitro* to further evaluated the effects of CPNE1 on ccRCC cell proliferation, migration, invasion. Mechanistically, CPNE1 might regulate ccRCC development through EGFR/STAT3 signaling.

## 2 Materials and Methods

### 2.1 Pan-cancer analysis

The expression of CPNE1 in various cancers, including KIRC (kidney renal clear cell carcinoma), was analyzed using the TIMER (https://cistrome. shinyapps. io/timer/) database based on The Cancer Genome Atlas (TCGA) data (Li et al., 2017a). We used TISIDB (http://cis.hku.hk/TISIDB/) database to analyze the relationships between CPNE1 expression and overall survival (OS), stage and grade in pan-cancer (Ru et al., 2019).

### 2.2 Analysis of CPNE1 mRNA expression, diagnostic and prognostic value in ccRCC

RNA-sequencing (RNA-seq) data and patients’ clinical information of TCGA-KIRC cohort were acquired from the TCGA database (https://portal.gdc.cancer.gov/). E-MTAB-1980 cohort was abtained from the ArrayExpress database (https://www.ebi.ac.uk/arrayexpress/experiments/E-MTAB-1980/) (Sato et al., 2013). GSE40435 and GSE53757 were served as validation cohorts. The potential value of CPNE1 in ccRCC diagnosis was detected with receiver operator characteristic (ROC) curve (Mandrekar, 2010). And Kaplan-Meier, univariate and multivariate analyses were used for prognostic prediction. Kaplan-Meier analysis using the optimum cut-off value determined by X-tile software (Camp et al., 2004). “rms” package of R software was used to establish the nomogram.

### 2.3 Co-expression analyses and functional enrichment analyses

Co-expressed genes with CPNE1 were identified using Pearson’s correlation analysis in LinkedOmics (http://www.linkedomics.org) database (Vasaikar et al., 2018). The results were showed *via* volcano plot and heatmaps. Moreover, we verified the results in the cBioPortal database (http://cbioportal.org) (Cerami et al., 2012). MiRNA-target enrichment was also performed by LinkedOmics. Functional enrichment analysis was carried out *via* the Metascape online database (http://metascape.org/) (Zhou et al., 2019).

### 2.4 Tumor immunology analysis

Stromal, immune, and estimate scores were calculated based on the ESTIMATE algorithm, which was generated from the expression data in the TCGA-KIRC dataset (Yoshihara et al., 2013). CIBERSORT provides a deconvolution algorithm that is used to calculate the fractions of the 22 types of tumor infiltrating immune cells (TIICs) (Newman et al., 2015). And we applied CIBERSORT algorithm to estimate the fractions of immune cell types between high- and low-expression groups. We achieved 47 immune checkpoint genes from a literature review (Supplementary Table S1 **)**. Subsequently, we analyzed the correlation between CPNE1 and the expression of these immune checkpoint genes in ccRCC using the “limma” package and Pearson test (Ritchie et al., 2015). Moreover, computational methods were used to evaluate the relationship between CPNE1 expression pattern and immunotherapy effect in ccRCC. The tumor immune dysfunction and exclusion (TIDE) algorithm was also used to predict the immunotherapy response. TIDE is a computing architecture developed to evaluate response of each sample to PD-1/PD-L1 and CTLA4 inhibitors based on gene expression profile (Jiang et al., 2018b).

### 2.5 Cell culture and reagents

Renal epithelial cell HK-2, human ccRCC cell line 786-O, OSRC, Caki-2, ACHN and A-498 were obtained from Department of Urology (Tongji Hospital, Wuhan, China). All cell lines were cultured in Dulbecco’s modified Eagle’s medium (DMEM) (HyClone, Logan, UT) containing 10% fetal bovine serum (FBS) (Gibco, Carlsbad, United States). Cells were incubated in a humidified incubator with 5% CO_2_ at 37°C.

### 2.6 Cell transfection

Cells were seeded in 6-well plates and incubated overnight to 70% confluent at the time of transfection. CPNE1-siRNA (General Biol, Anhui, China) and overexpression plasmid (GeneChem, Shanghai, China) were transfected into ccRCC cell lines using Lipofectamine 3,000 Transfection Reagent (Invitrogen, Carlsbad, United States) according to the instructions.

### 2.7 RNA extraction and real-time PCR (RT–PCR)

Total RNA was extracted with Trizol reagent (Invitrogen, Carlsbad, United States). cDNA synthesis was carried out using HiScript II Q RT SuperMix (Vazyme Biotech, Nanjing, China). Real-time PCR analysis was conducted using ChamQ Universal SYBR qPCR Master Mix (Vazyme Biotech, Nanjing, China) with a 7,900 Real-time PCR system (Applied Biosystems, Foster City, United States). The relative mRNA expression of indicated genes were calculated using delta-delta Ct (2^−ΔΔCT^) method (Livak and Schmittgen, 2001). The primer sequences are available in (Supplementary Table S2).

### 2.8 Cell counting Kit‐8 (CCK8) assay

2 × 10^3^ transfected cells/well were plated into 96-well plates with 100 μL medium. After the cells adhered to the wall, the supernatants were removed, and serum-free medium containing 10% CCK8 was added to each well for another 2 h incubation. Then the OD value at 450 nm for 0, 24, 48, and 72 h were measured through microplate reader (BioTek, Winooski, United States), respectively.

### 2.9 Wound healing assay

The cells were seeded in six-well plates with DMEM containing 10% FBS to full confluence. Subsequently, the cell monolayer was scratched with a 200 μL pipette tip, washed with PBS twice and cultured in serum-free medium. Wound closures were photographed by a microscope at 0 h and 24 h.

### 2.10 Transwell assay

Transwell assays were conducted using Transwell chambers (8-μm pores; Corning Costar, Corning, NY, United States) in 24-well plates. For cell invasion assays, the filters were pre-coated with a Matrigel matrix (BD Science, Sparks, MD, United States). 1 × 10^5^ cells in 100 uL serum-free DMEM medium were seeded in the upper chamber, and 600 μL DMEM medium containing 20% FBS was added to the lower chamber, followed by cultured in the incubator for 24 h. Then, the chambers were washed by PBS twice, fixed by paraformaldehyde for 15 min and stained with 0.5% crystal violet for 10 min.

### 2.11 Western blotting analysis

Total cellular protein was extracted with RIPA lysis buffer (Servicebio, Wuhan, China) containing a proteinase inhibitor cocktail and phosphorylase inhibitor (Servicebio, Wuhan, China). The protein samples were subsequently mixed with 5 × SDS-PAGE loading buffer and boiled at 95°C for 5 min. 30 ug protein samples were subjected to 10% SDS polyacrylamide gel electrophoresis (SDS-PAGE) and transferred to a PVDF membrane (Millipore, Billerica, United States). The membrane was blocked with 5% skimmed milk at room temperature for 1 h and incubated with the following primary antibodies overnight at 4°C: anti-GAPDH antibody (Proteintech, 6,004, 1:20,000), anti-CPNE1 antibody (Abcam, Cambridge, United Kingdom, ab155675, 1:1000), anti-STAT3 (Cell Signaling Technology, Danvers, United States, #9139, 1:1000), anti-p-STAT3 (Cell Signaling Technology, Danvers, United States, #9145, 1:1000), anti -EGFR (Abmart, Shanghai, China, T55112, 1:5000), anti-phospho-EGFR (Cell Signaling Technology, Danvers, United States, #3777, 1:1000). After washing with TBST three times for 10 min each, the membranes were incubated with secondary antibodies (Promoter, Wuhan, China, 1:5000) at room temperature for 1 h. Finally, West Pico Plus Chemiluminescent Substrate (Thermo Fisher Scientific, Waltham, United States) was used to visualize the protein bands.

### 2.12 Statistical analysis

All statistical analyses were processed using R software (v.4.1.2) and Prism 8.0. The Wilcoxon test or Kruskal–Wallis test was used to analyze the relationship between clinicopathological characteristics and the CPNE1 expression. Correlation analyses were performed by Pearson correlation test. For experimental data, all data were expressed as mean ± SD. Significant differences between groups were determined using the two‐tailed Student’s t-test or one‐way ANOVA. *p* < 0.05 was considered significant. *p < 0.05, **p < 0.01, ***p < 0.001.

## 3 Results

### 3.1 Expression and prognostic value of CPNE1 in human pan-cancer

**FIGURE 1:**
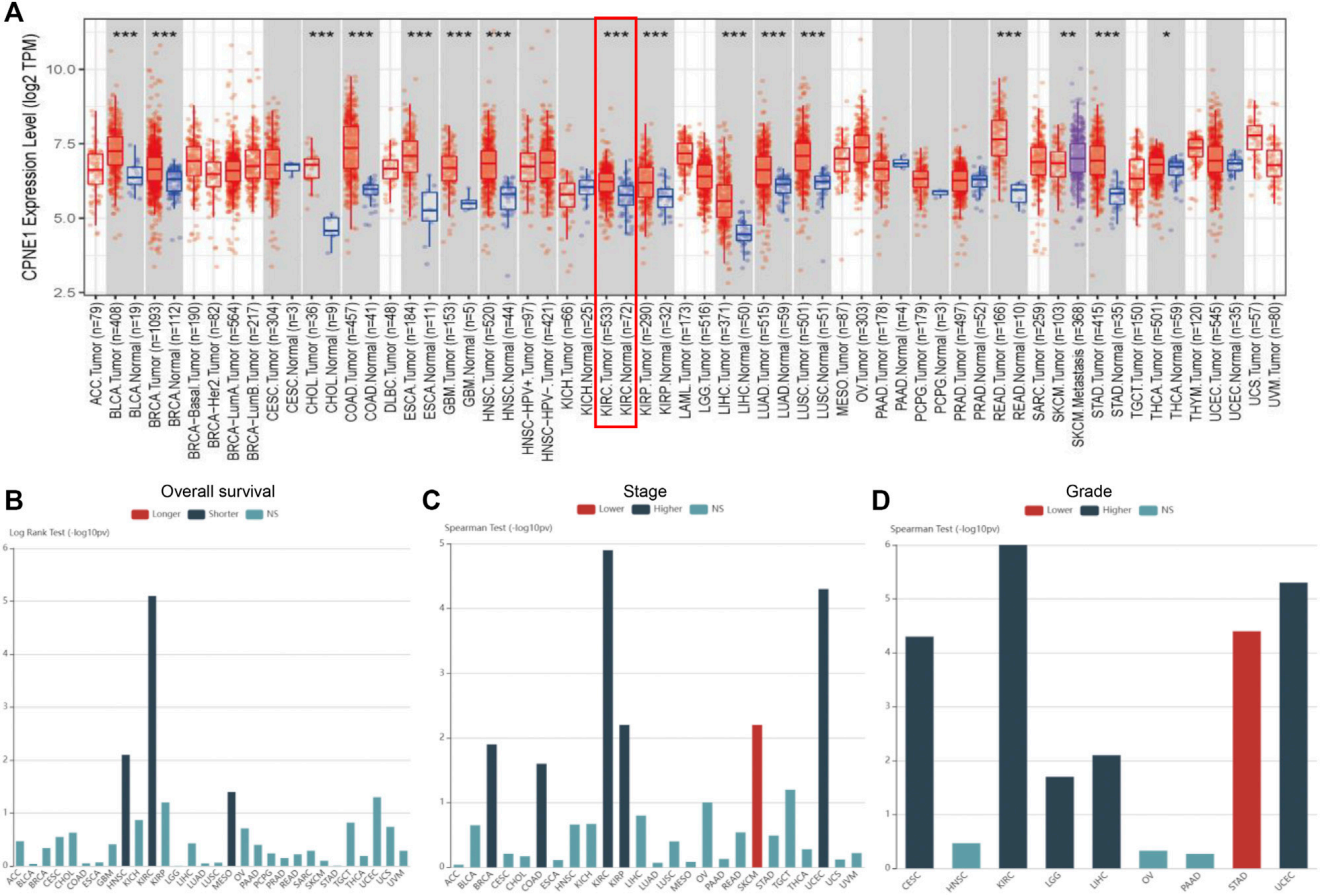
Pan-cancer analysis. **(A)** CPNE1 was aberrantly expressed among multiple cancer types. **(B)** High expression of CPNE1 predicted worse prognosis in HNSC, KIRC, and MESO. **(C)** High expression of CPNE1 was associated with advanced stage in BRCA, COAD, KIRC, KIRP, UCEC. **(D)** High CPNE1 expression was correlated with high grade in CESC, KIRC, LGG, LIHC, UCEC. *, *p* < 0.05; **, *p* < 0.01; ***, *p* < 0.001 (Supplementary Table S3 showed a complete list of the TCGA cancer-type abbreviations).

TIMER was used to find out differences in mRNA expression of CPNE1, between tumor and normal tissue, in multiple cancers. As shown in Figure 1A, CPNE1 was significantly upregulated in 15 tumor types including KIRC. Subsequently, we carried out a comprehensive expression-clinical analysis of CPNE1 in 33 types of TCGA cancer through LinkedOmics. As shown in Figure 1B, in HNSC (head and neck squamous cell carcinoma), KIRC (kidney renal clear cell carcinoma) and MESO (Mesothelioma), patients with high CPNE1 expression possessed shorter overall survival times. In five types including KIRC, patients with high CPNE1 expression related to higher stage and grade (Figures 1C, D). (Supplementary Table S3 showed a complete list of the TCGA cancer-type abbreviations)

### 3.2 Expression and prognostic significance of CPNE1 in ccRCC

**FIGURE 2:**
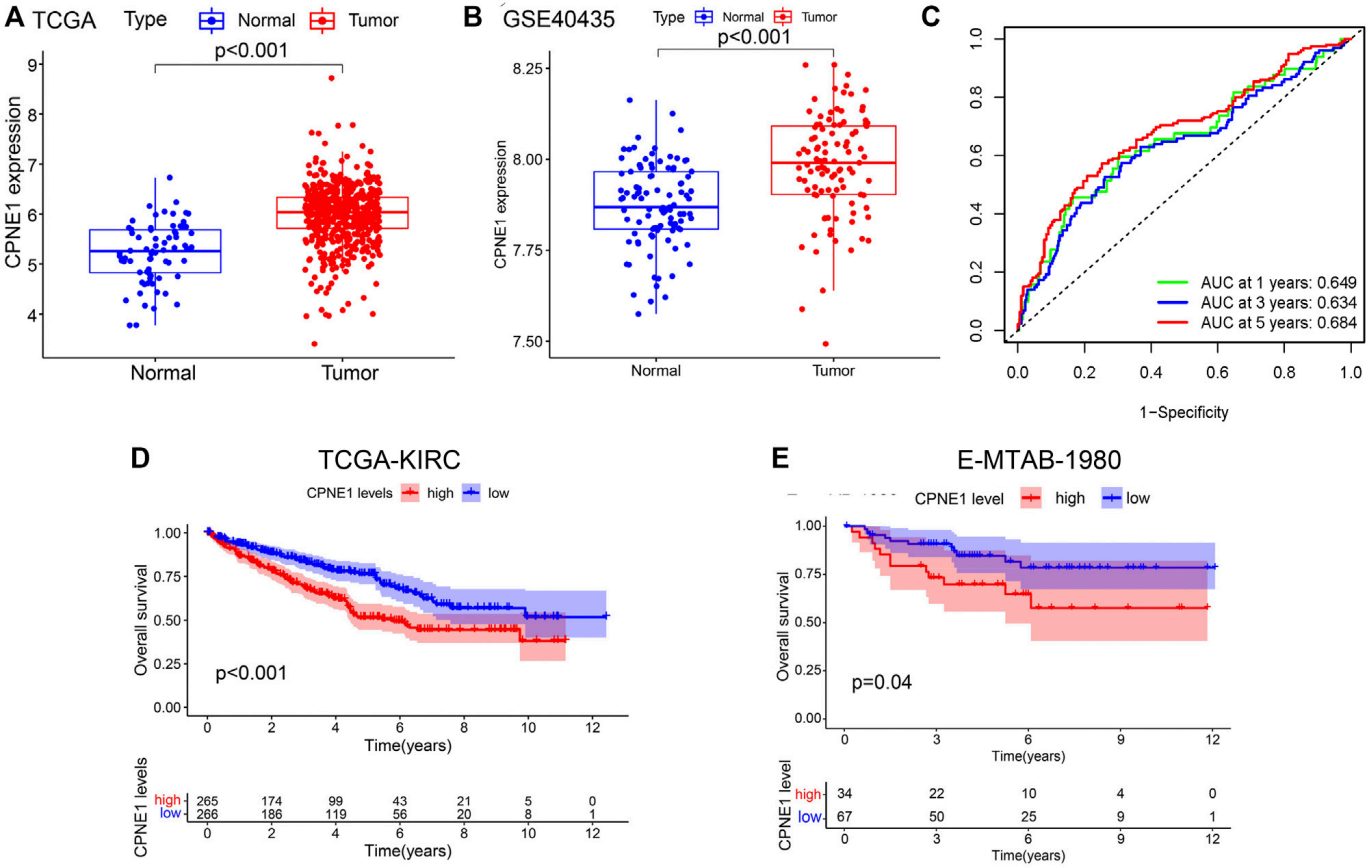
CPNE1 mRNA expression, diagnostic and prognostic value in ccRCC. **(A)** CPNE1 was significantly overexpressed in tumors compared with the adjacent normal tissue in TCGA-KIRC dataset (*p* < 0.001). **(B)** CPNE1 was significantly overexpressed in tumors compared with the adjacent normal tissue in GSE40435 (*p* < 0.001). **(C)** ROC curve showed that the prediction of the AUC of the 1-year, 3-year and 5-year OS were 0.649, 0.634 and 0.684 respectively. **(D)** Kaplan–Meier plot demonstrated a correlation between poor prognosis and the high CPNE1 expression in TCGA-KIRC dataset (*p* < 0.001). **(E)** Kaplan–Meier plot demonstrated a correlation between poor prognosis and the high CPNE1 expression in E-MTA-1980 dataset (*p* = 0.04).

CPNE1 was significantly increased in KIRC tissue compared with adjacent non-cancerous tissue samples both in TCGA and GSE40435 (Figures 2A, B **)**. The area under the ROC curve (AUC) of CPNE1 expression for OS was 0.649 at 1 year, 0.634 at 3 years, and 0.684 at 5 years (Figure 2C). Kaplan-Meier analysis revealed that patients with higher CPNE1 expression exhibited worse outcome than those with lower CPNE1 expression both in TCGA and E-MTAB-1980 dataset (Figures 2D, E).

### 3.3 Associations between CPNE1 expression and clinicopathological characteristics in ccRCC patients

**FIGURE 3:**
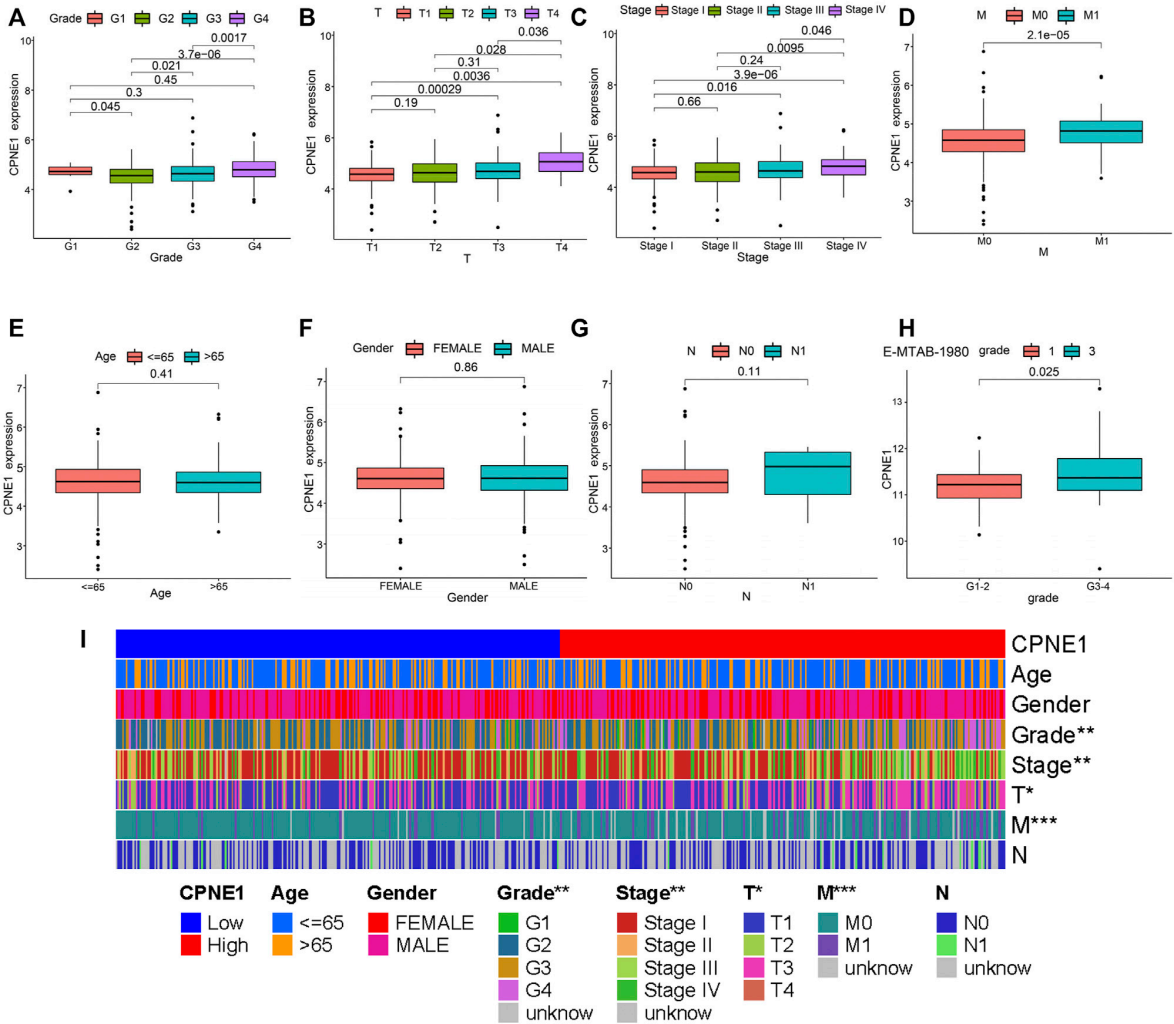
CPNE1 was correlated with various clinicopathological parameters in ccRCC. High CPNE1 expression was positively correlated with **(A)** tumor grade, **(B)** invasion range, **(C)** stage and **(D)** distant metastasis. CPNE1 was not associated with **(E)** age, **(F)** gender and **(G)** lymph node metastasis in TCGA-KIRC dataset. **(H)** High CPNE1 expression was associated with advanced tumor grade in E-MTAB-1980 dataset. **(I)** Heatmap showed the association of CPNE1 and clinicopathological features. *, *p* < 0.05; **, *p* < 0.01; ***, *p* < 0.001.

Based on the CPNE1 expression data and clinical information from TCGA, a total of 537 ccRCC patients were analyzed. The results showed that the expression of CPNE1 was significantly correlated with grade (G2 vs. G3, *p* = 0.021; G2 vs. G4, *p* < 0.01; G3 vs. G4, *p* < 0.01), invasion range (T1 vs. T3, *p* < 0.01; T1 vs. T4, *p* < 0.01; T2 vs. T4, *p* = 0.028; T3 vs. T4, *p* = 0.036), stage (stage I vs. stage III, *p* = 0.016; stage I vs. stage IV, *p* < 0.01; stage II vs. stage IV, *p* < 0.01; stage III vs. stage IV, *p* = 0.046) and distant metastasis (M0 vs. M1, *p* < 0.01), while not correlated with gender (*p* = 0.41), age (*p* = 0.86), and lymph node metastasis (*p* = 0.11) (Figures 3A–G). Moreover, we used E-MTAB-1980 dataset to verify the relationship between CPNE1 expression and grade (Figure 3H), GSE53757 to verify the relationship between CPNE1 expression and stage (Supplementary Figure S1), which were consistent with the results in TCGA. The distribution of clinicopathological features in high- and low expression of CPNE1 groups were further visualized by R package with heatmaps (Figure 3I).

### 3.4 CPNE1 is an independent prognostic factor in ccRCC

**FIGURE 4:**
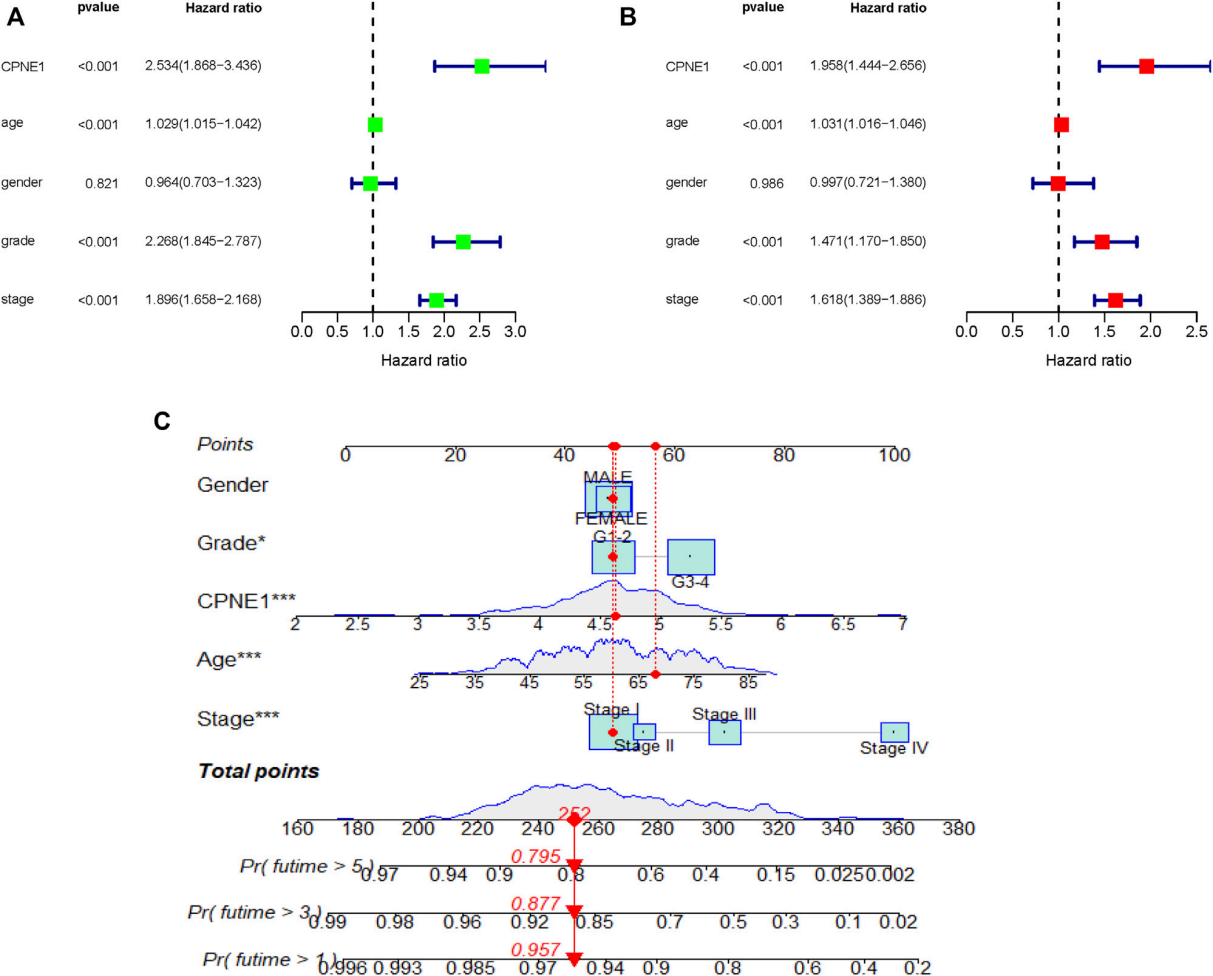
Independent prognostic analysis. **(A)** Univariate analysis showed that CPNE1, age, grade and stage were related to OS (*p* < 0.01). **(B)** Multivariate analysis showed that CPNE1, age, grade and stage were strong independent prognostic factors. **(C)** Nomogram represented the multivariate model. *, *p* < 0.05; **, *p* < 0.01; ***, *p* < 0.001.

Univariate and multivariate Cox regression analyses were performed to determine independent prognostic factors. The results showed that CPNE1 (HR = 1.958, 95% CI: 1.444–2.656, *p* < 0.001), age (HR = 1.031, 95% CI: 1.016–1.046, *p* < 0.001), grade (HR = 1.471, 95% CI: 1.170–1.850, *p* < 0.001) and stage (HR = 1.618, 95% CI: 1.389–1.886, *p* < 0.001) were independent prognostic factors for ccRCC (Figures 4A, B). In addition, to predict the prognosis of each patient, a nomogram integrated expression of CPNE1, gender, grade, age and stage was established (Figure 4C).

### 3.5 Co-expression genes of CPNE1 and enrichment analysis in patients with ccRCC

**FIGURE 5:**
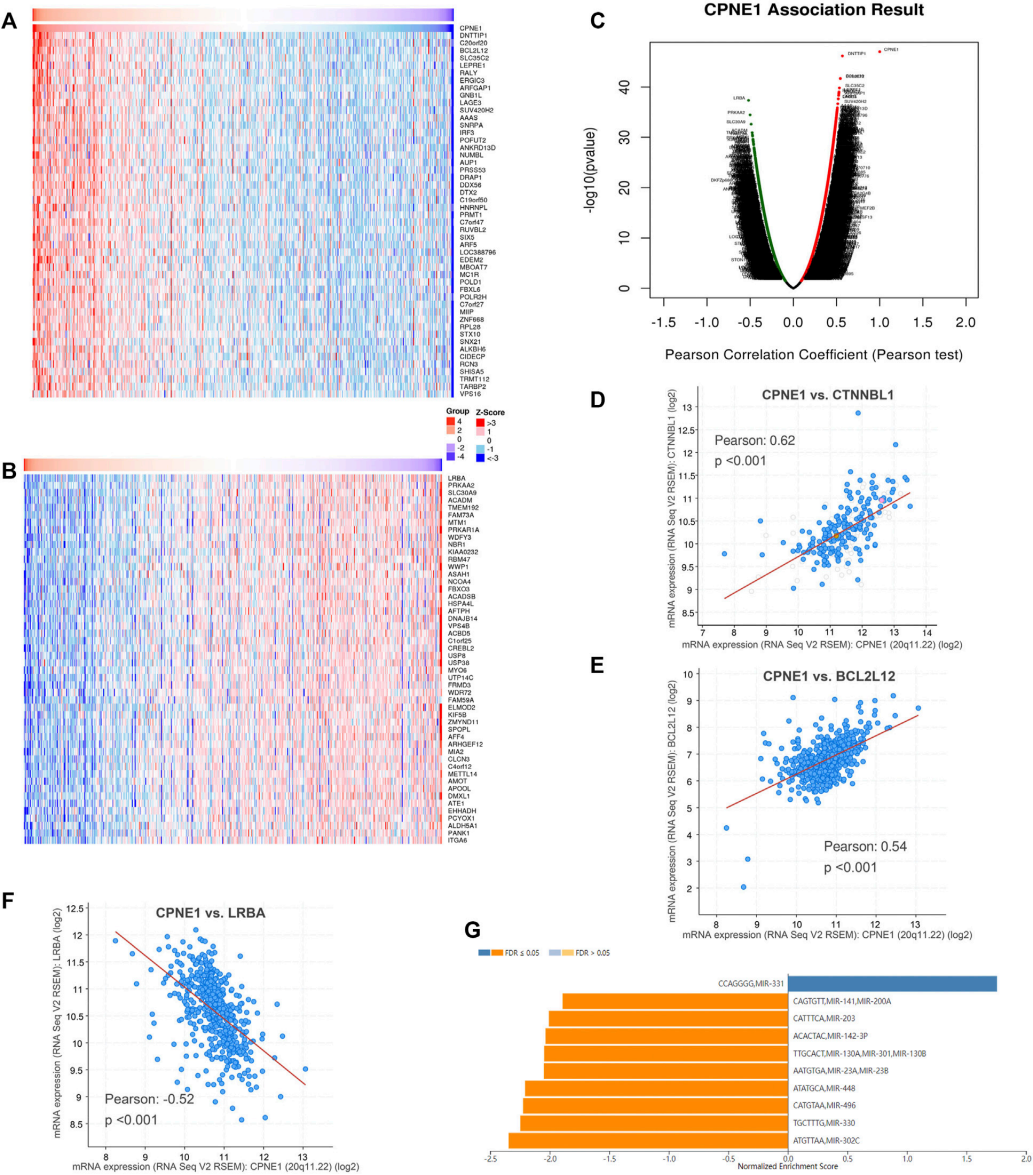
Co-expression genes of CPNE1 and miRNA-target enrichment analysis. **(A)** Heatmaps showed top 50 genes positively correlated with CPNE1. **(B)** Heatmaps showed top 50 genes negatively correlated with CPNE1. **(C)** Volcano plot showed the CPNE1 highly correlated genes. Positive correlations are displayed in red and negative correlations in green color. The top 3 genes co-expressed with CPNE1 were verified in the cBioPortal database. **(D)** CTNNBL1 was positively correlated with CPNE1 (R = 0.62, *p* < 0.001). **(E)** BCL2L12 was positively correlated with CPNE1 (R = 0.54, *p* < 0.001). **(F)** LRBA was negatively correlated with CPNE1 (R = −0.52, *p* < 0.001). **(G)** miRNA-target enrichment of CPNE1 based on LinkedOmics. R, Pearson correlation coefficient.

**FIGURE 6:**
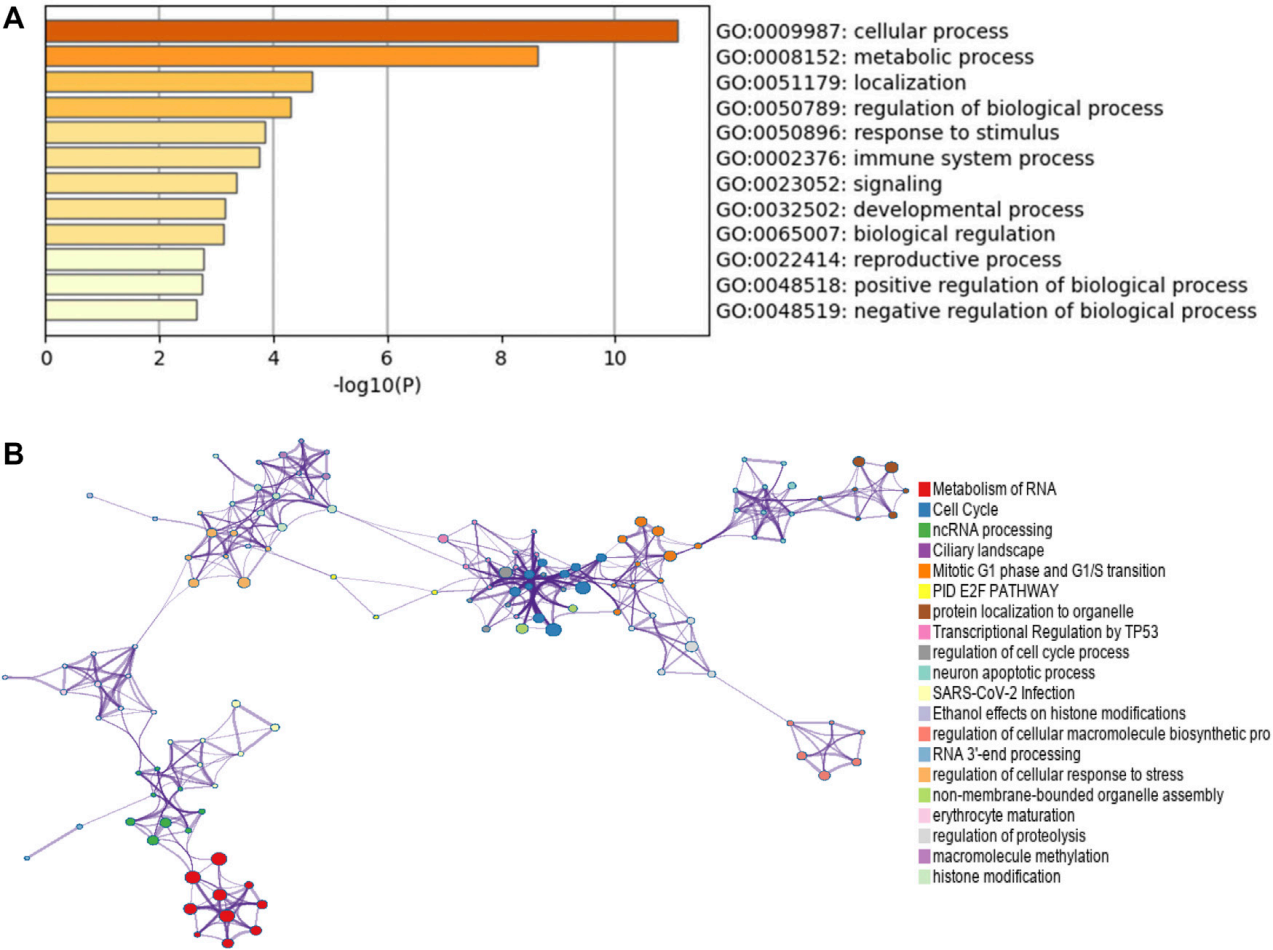
Function enrichment analysis of co-expressed genes with CPNE1 by Metascape. **(A)** GO annotation showed that CPNE1 was related to the GO term “cellular process”, “metabolic process” and “regulation of biological process.” **(B)** KEGG pathway showed that CPNE1 was related to “metabolism of RNA”, “Cell Cycle” and “ncRNA processing”.

To explore the potential genes co-expressed with CPNE1, we used the function module of LinkedOmics database. The CPNE1 association results were analyzed and visualized in the volcano plot (Figure 5A). The heatmaps were used to identify the top 50 significant positively/negatively correlated genes with CPNE1, as shown in Figures 5B, C. Subsequently, we employed the co-expression module of the cBioPortal database to verify the results, which showed that DNTTIP1(Pearson: 0.62, *p* < 0.01) and BCL2L12 (Pearson: 0.54, *p* < 0.01) were positively related to CPNE1, and LRBA (Pearson: −0.52, *p* < 0.01) was negatively related to CPNE1 (Figures 5D–F). To further explore the targets of CPNE1 in ccRCC, we analyzed miRNA network of co-expressed genes with CPNE1. The top five most significant miRNAs related to CPNE1 expression were miR-302c, miR-330, miR-496, miR-448, and miR-23 (Figure 5G). Using Metascape database, we found that these co-expressed genes were primarily involved in metabolism of RNA, ncRNA processing, cell cycle, E2F pathway and immune system process (Figures 6A, B).

### 3.6 Correlation between CPNE1 expression with immune cell infiltration, immune modulators and immune therapy

**FIGURE 7:**
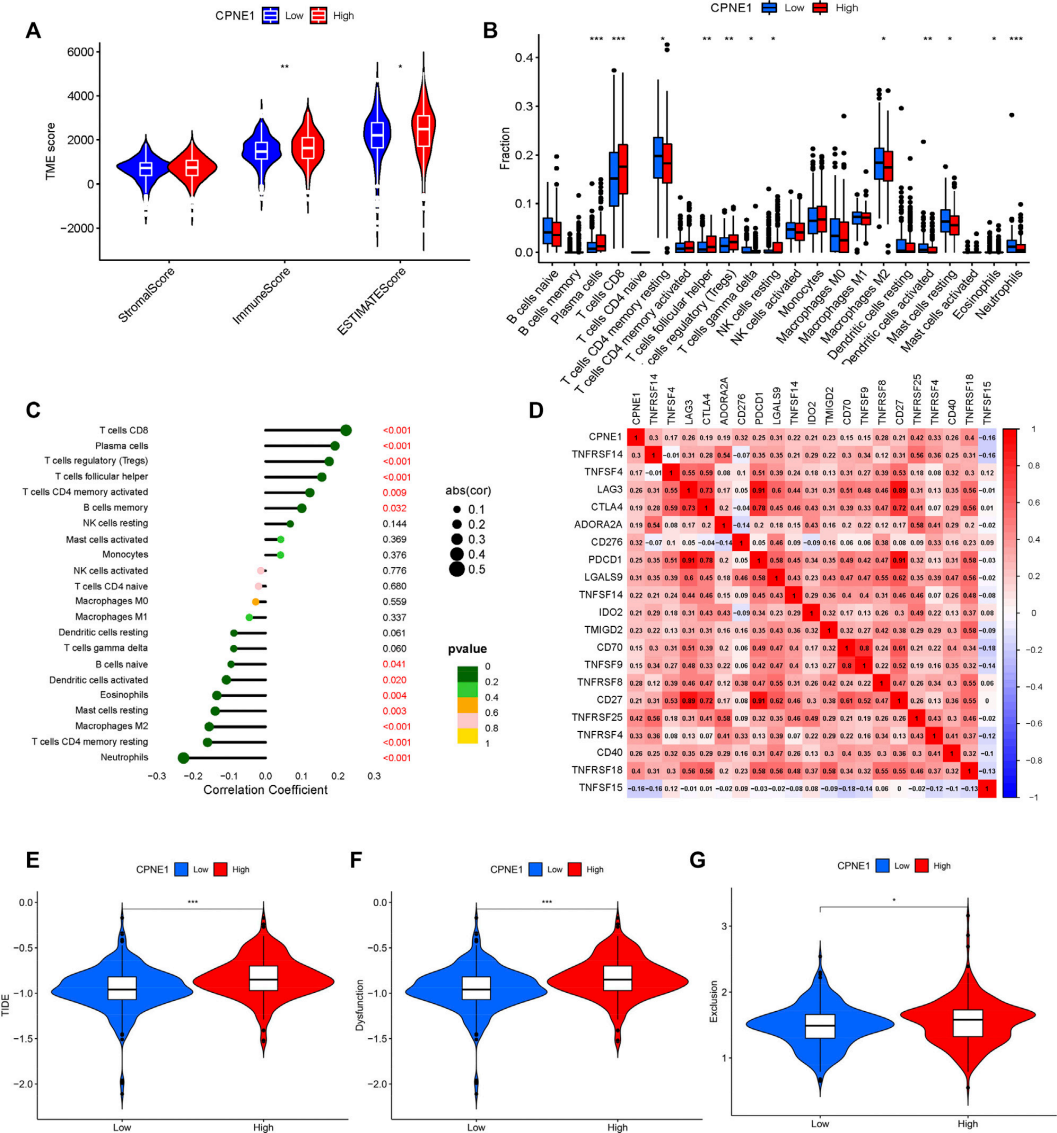
Tumor immunology analysis. **(A)** CPNE1 was positively correlated with immune and estimate scores. **(B)** CPNE1 high- and low-expression groups displayed different fraction of immune cell types. The x-axis represents the types of immune cell. The y-axis represents the proportion of each immune cell type. **(C)** CPNE1 was significantly related to the proportions of multiple immune cell types. **(D)** Heatmap showed CPNE1 was associated with multiple immune checkpoint members. Heatmap colors: correlation (cor) coefficient. CPNE1 high -expression groups displayed higher **(E)** TIDE score, **(F)**T cell dysfunction score, and **(G)** T cell exclusion score. *, *p* < 0.05; **, *p* < 0.01; ***, *p* < 0.001.

In the tumor microenvironment, the presence of immune cells plays a crucial role in cancer development. To understand the microenvironment of ccRCC, stromal, immune, and estimate scores were calculated by applying the ESTIMATE algorithm to the expression data downloaded from TCGA dataset. The results showed that CPNE1 expression was significantly related to immune and estimate scores, but not to stromal score (Figure 7A), implying that a higher immune cell content in the high CPNE1 expression group. Tumor-infiltrating immune cells play indispensable roles during cancer development (Gajewski et al., 2013). We then used CIBERSOR to calculate 22 kinds of infiltrating immune cells in patients with different CPNE1 expression level. As shown in Figures 7B, C, the high CPNE1 expression group exhibited higher infiltrations of immune cells, such as CD8^+^ T cells, plasma cells, regulatory T cells, follicular helper T cells and CD4 memory activated T cells, exhibited lower infiltrations of neutrophils, CD4 memory resting T cells and M2 macrophages. Immunomodulators are involved in other intrinsic immune escape mechanisms and play an essential role in cancer immunotherapy (Tang et al., 2018b). Meanwhile, the high CPNE1 expression group exhibited elevated expression levels of CD8^+^ T cell exhaustion markers (Díaz-Montero et al., 2020), including CTLA4, PDCD1, and LAG3 when compared to the low CPNE1 expression group (Figure 7D). T cell exhaustion has been extensively described as a mechanism for inhibiting the cell proliferation and the cytotoxic potential of CD8+T cells (Zajac et al., 1998; Jansen et al., 2019). Thorsson et al. implemented a pan-cancer classification identifying six immune subtypes (C1–C6), revealed that the immune subtype C1 was featured by a high proliferation rate and a poor prognosis than the other immune subtypes, while C3 and C5 had better outcome and represent immune equilibrium (Thorsson et al., 2018). It is worth noting that C1 was enriched in high CPNE1 expression group, while C3 and C5 were enriched in low CPNE1 expression group (Supplementary Figure S2). Overall, although the high CPNE1 expression group exhibited higher immune infiltrations, there might exist extrinsic immune escape mechanisms that leading to worse prognosis. The low CPNE1 expression group may represent immune equilibrium and result in a better outcome. We then applied the TIDE model to predict the efficacy of immunotherapy. The results showed that the high CPNE1 expression group had higher TIDE scores than the low expression group (Figure 7E), which indicated a higher potential for immune evasion and a fewer benefit from immunotherapy in the high CPNE1 expression group. Furthermore, the scores of T-cell dysfunction and T-cell exclusion (Figures 7F, G) were significantly different in these two groups. The above results suggested that the expression of CPNE1 was related to the immune escape and immunotherapy responses.

### 3.7 The expression level of CPNE1 in ccRCC cell lines and construction of knockdown cell lines

**FIGURE 8:**
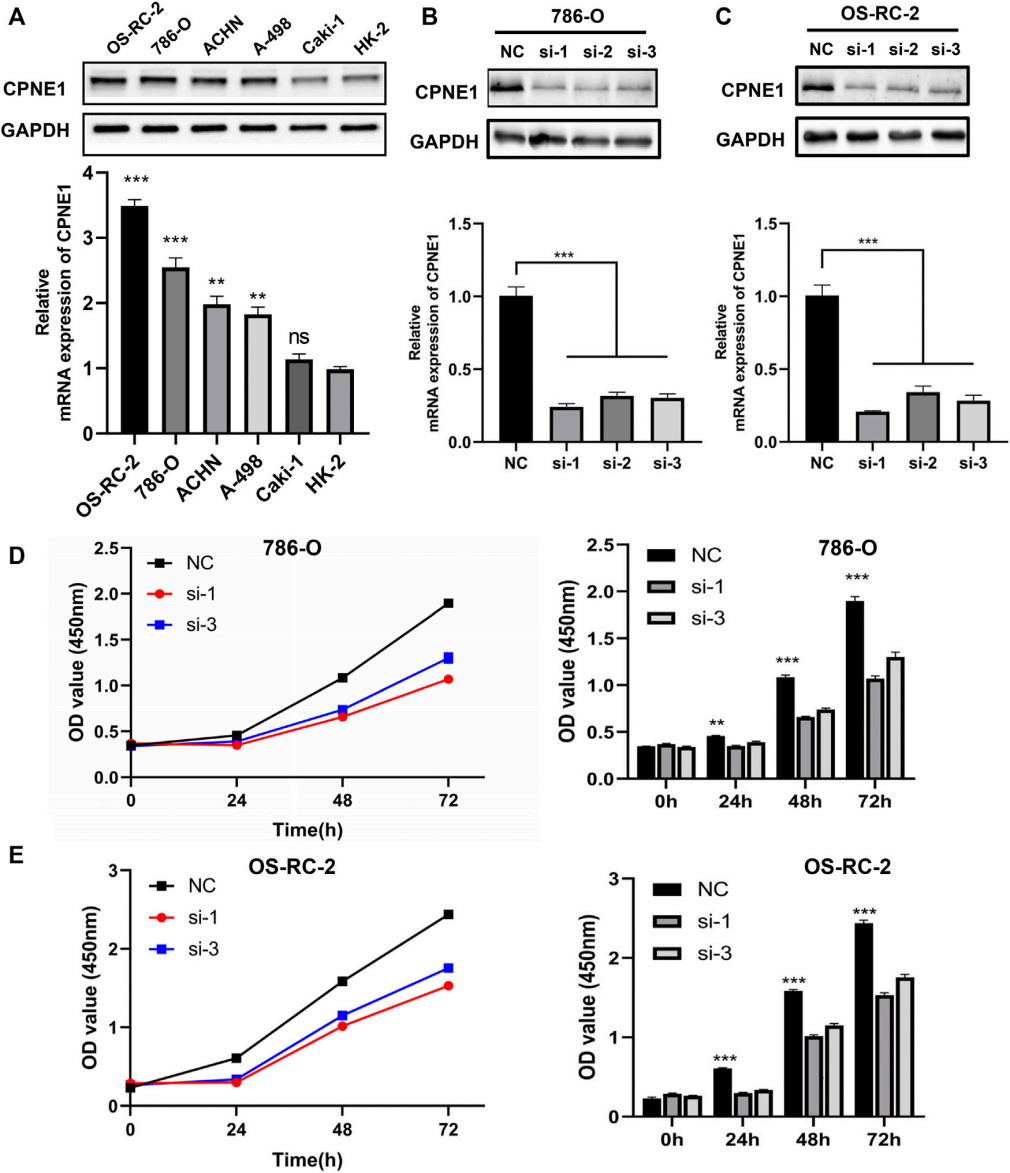
Construction of CPNE1 knockdown cell lines and cell proliferation assays. **(A)** The mRNA and protein expression level of CPNE1 in ccRCC cell lines was generally higher than that in renal epithelial cell. **(B)** Successful knockdown by the siRNA was confirmed by RT-PCR and WB in 786-O cells. **(C)** Successful knockdown by the siRNA was confirmed by RT-PCR and WB in OS-RC-2 cells. **(D)** Knockdown of CPNE1 inhibited 786-O cells proliferation. **(E)** Knockdown of CPNE1 inhibited OS-RC-2 cells proliferation. **, *p* < 0.01; ***, *p* < 0.001. NC: Negative control. Si: ccRCC cells transfected with CPNE1-small interfering RNA.

Based on the above results, a series of gain-of-function and loss-of-function experiments were conducted to further determine the potential role of CPNE1 in ccRCC cells. We performed RT-PCR and WB in renal epithelial cell (HK-2), and five human ccRCC cell lines (786-O, OS-RC-2, Caki-2, ACHN and A-498) to verify CPNE1 mRNA. Compared with HK-2 cell, the protein and mRNA expression level of CPNE1 in 786-O, OS-RC-2, ACHN and A-498 was generally upregulated, which suggested the expression level of CPNE1 in ccRCC cell lines was generally higher than that in renal epithelial cell (Figure 8A). The result was consistent with our bioinformatic analysis.

The CPNE1 expression in mRNA level was the highest in OS-RC-2 cell, followed by 786-O, ACHN, A-498 and caki-2 cells. Therefore, we chose OS-RC-2 and 786-O cells to construct CPNE1-knockdown cell models for subsequent experiments. OS-RC-2 and 786-O cells were transiently transfected with small interfering RNA (siRNA) against CPNE1 or control siRNA. Knockdown efficiency was detected 24 h later by RT-qPCR and 48 h later by Western blot. Compared with the control siRNA, CPNE1 expression levels were significantly reduced by siRNA transfection both in OS-RC-2 and 786-O cells (Figures 8B, C). Si-CPNE1-1 and si-CPNE1-3 presented better knockdown efficiency and therefore were chosen for following experiments. We further examined the mRNA expression of all other eight copines family members and verified that the silence of CPNE1 did not affect the expression of CPNE2-CPNE9 (Supplementary Figure S3).

### 3.8 CPNE1 knockdown suppresses ccRCC cell proliferation, migration and invasion *in Vitro*

CCK8 assay was performed to evaluate the cell proliferation differences between control siRNA and CPNE1-siRNA transfected groups. The results revealed that the proliferation of 786-O and OS-RC-2 cells was significantly decreased after CPNE1 knockdown (Figures 8D, E).

**FIGURE 9:**
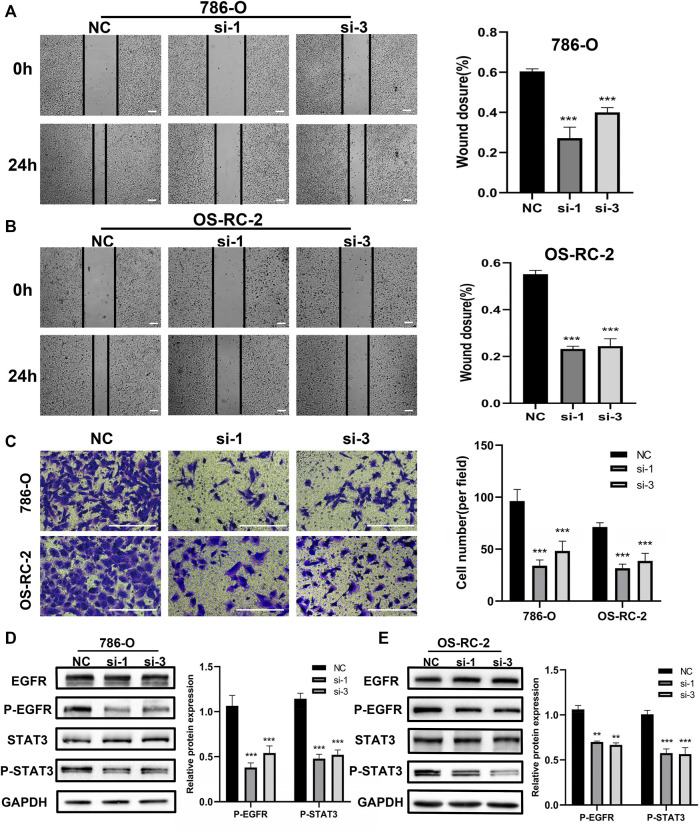
Biological functions of CPNE1 knockdown *in vitro*. **(A)** Knockdown of CPNE1 decreased migration of 786-O cells (scale bar: 50 μm). **(B)** Knockdown of CPNE1 decreased migration of OS-RC-2 cells (scale bar: 50 μm). **(C)** CPNE1 knockdown significantly inhibited 786-O and OS-RC-2 cells invasion (scale bar: 200 μm). **(D)** EGFR and STAT3 phosphorylation were inhibited by the knockdown of CPNE1 in 786-O cells. **(E)** EGFR and STAT3 phosphorylation were inhibited by the knockdown of CPNE1 in OS-RC-2 cells. NC: Negative control. Si: ccRCC cells transfected with CPNE1-small interfering RNA. **, *p* < 0.01; ***, *p* < 0.001.

The above bioinformation analysis showed that patients with distant metastasis had higher CPNE1 expression than patients without distant metastasis. In addition, elevated CPNE1 was also related to late stage in ccRCC. Therefore, we next detected the cytological effect of CPNE1 on the migration ability of ccRCC cells using a scratch-healing assay. The results showed CPNE1 knockdown significantly inhibited 786-O and OS-RC-2 cell migration (Figures 9A, B). Transwell invasion assay revealed that CPNE1 silencing also decreased the invasiveness of 786-O and OS-RC-2 cells (Figure 9C).

### 3.9 CPNE1 regulates EGFR/STAT3 pathway in ccRCC

EGFR signaling play an important role in tumorigenesis and progression (Tomas et al., 2014). Previous studies showed that CPNE1 is a critical factor in the tumorigenesis of lung cancer and that its mechanism involves the EGFR signaling pathway (Tang et al., 2018a; Wang et al., 2022). Therefore, we detected the effect of CPNE1 on the regulation of EGFR signaling pathway in ccRCC. Protein expression levels of EGFR and p-EGFR, STAT3 and p-STAT3 were detected by Western blot analysis. In comparison with the negative control (NC) group, the CPNE1-siRNA groups showed significantly decreased expression levels of p-EGFR and p-STAT3 (*p* < 0.01), while the total EGFR and STAT3 protein expression level remained unchanged (Figures 9D, E). These results indicated that CPNE1 might activate the EGFR/STAT3 signaling pathway to promote ccRCC cell growth.

### 3.10 Effects of CPNE1 overexpression on ccRCC cells

**FIGURE 10:**
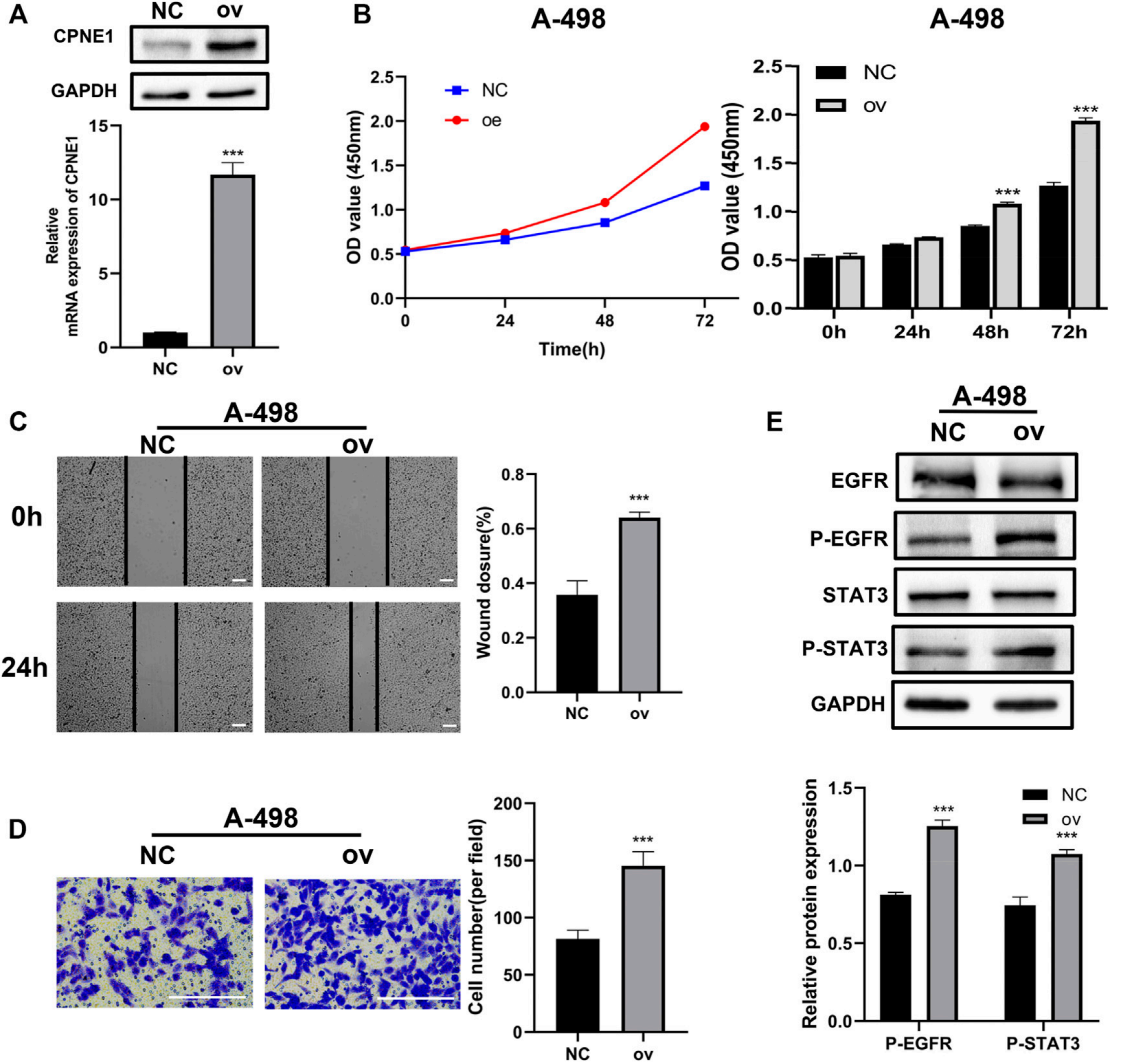
Biological functions of CPNE1 overexpression *in vitro*. **(A)** The successful construction of CPNE1 overexpression (ov) plasmid was validated *via* RT-PCR and WB in A-498 cells. **(B)** Overexpression of CPNE1 promoted proliferation in A-498 by CCK8 assays. **(C)** Scratch-wound healing assays demonstrated that migration abilities of A-498 cells were enhanced by overexpression of CPNE1 (scale bar: 50 μm). **(D)** Invasive capacities of A-498 cells were enhanced by CPNE1 overexpression (scale bar: 200 μm). **(E)** EGFR and STAT3 phosphorylation were activated by the overexpression of CPNE1 in A-498 cells. NC: negative control. ***, *p* < 0.001.

To further characterize the function of CPNE1 in ccRCC cells, A-498 cell was used for subsequent CPNE1 overexpression experiments. Transfection efficiency of overexpression plasmid for CPNE1 was confirmed by RT-qPCR and Western blot analysis. Compared to the NC group, the mRNA and protein expression levels of CPNE1 in transfected cell were both significantly elevated (Figure 10A). CCK-8 assays showed an increased cell growth in CPNE1-overexpressing A-498 cell (Figure 10B). Next, we investigated the effects of CPNE1 overexpression on ccRCC cell invasion and migration ability using wound healing assay and transwell assay. We observed that the CPNE1-overexpressing A-498 cells migration and invasion abilities were significantly stronger than that of the NC group (Figures 10C, D). In addition, the protein expression level of p-EGFR and p-STAT3 in CPNE1-overexpressing A-498 cell was markedly increased, while the expression level of total EGFR and STAT3 exhibited no noticeable changes in A-498 cell (Figure 10E).

## 4 Discussion

KIRC is one of the most common malignancies threatening public health and posing significant global health issues. Despite aggressive treatment regimens, patient prognosis is often poor and marked by tumor recurrence and/or metastasis (Barata and Rini, 2017). The occurrence and development of ccRCC is a complex process involving multiple signaling pathways (Jonasch et al., 2021). Therefore, exploring the molecular mechanisms of malignant proliferation and metastasis of renal cancer cells is an effective approach for discovering new drug targets. In addition, this is very critical to the development of individualized and effective therapy strategy.

Copine 1 is one of the nine members of the Copine family (CPNE1–9). Copine 1, which encoded by CPNE1, is a calcium‐dependent phospholipid‐binding protein that plays an essential role in calcium‐mediated intracellular processes (Tomsig et al., 2004). CPNE1 tertiary structure folds into three distinct domains: the N-terminal two tandem C2 domains and the C-terminal one vWA domain (Perestenko et al., 2010). CPNE1 is a highly conserved protein and is ubiquitously expressed in various tissues, including brain, lung, prostate, liver, colon, kidney and heart (Tomsig and Creutz, 2000). CPNE1 was involved in intracellular signal transduction and membrane trafficking, and interact with intracellular proteins (Perestenko et al., 2010; Smith et al., 2010). A recent study indicated that CPNE1 might serve as an independent prognostic biomarker for ccRCC (Talaat et al., 2022). However, the biological function, molecular mechanisms and immune implication of CPNE1 in ccRCC remains unknown.

Accumulating evidence reveals a key role of CPNE1 in cancer progression and metastasis. CPNE1 is able to regulate glycolysis, and promote colorectal cancer cell growth and drug resistance through AKT-GLUT1/HK2 pathway (Wang et al., 2021). CPNE1 influenced cell proliferation, cytochrome c-mediated caspase cascade apoptosis and arrested cell cycle in gastric cancer *via* DDIT3-FOS-MKNK2 axis (Li et al., 2022). In addition, CPNE1 is highly expressed in prostate cancer. Its expression is positively correlated with advanced tumor stages, poor prognosis and TRAF2 expression (Liang et al., 2017). Consist with these studies, we demonstrated that CPNE1 was significantly upregulated in ccRCC tissues compared to adjacent normal tissues. Elevated CPNE1 expression was related to advanced tumor stage, histological grade, distant metastasis, and shorter survival time. Moreover, overexpression of CPNE1 promoted the proliferation, migration and invasion of ccRCC. These results demonstrated that CPNE1 might be a potential target for ccRCC treatment and also added to an emerging understanding of the biological functions of CPNE1 in ccRCC.

From the protein co-expression analysis, we found CTNNBL1, BCL2L12 and LRBA were most highly correlated with CPNE1. CTNNBL1 is overexpressed in ovarian cancer cell lines and related to poor prognosis. Besides, it increased ovarian cancer proliferation, migration and invasion (Li et al., 2017b). BCL2L12 (Bcl2-like 12) universally overexpressed in human glioma specimens and contributed to important disease characteristics, including resistance to chemotherapy-induced apoptosis (Stegh et al., 2007). High expression of BCL2L12 was related to unfavorable prognosis in nasopharyngeal carcinoma and might suggest a novel biomarker for predicting short-term relapse (Fendri et al., 2011). LRBA, a member of the BEACH-WD40 protein family, is essential for immune function. It had a role in regulating cell surface expression of CTLA4, a key inhibitor of T-cell activation and proliferation (Vardi et al., 2020). Further functional enrichment analyses showed that these co-expressed genes were primarily involved in metabolism of RNA, immune system process, ncRNA processing, regulation of cell cycle, E2F pathway and transcriptional regulation by TP53. CcRCC is known as a highly metabolic disease and metabolic changes provide the basis for progression to advanced ccRCC (Wettersten et al., 2017). Tp53 is known as an important cell cycle regulator and a tumor suppressor. A previous study reported the abilities of CPNE1 to stimulate the proliferation and cell migration mediated by AKT/P53 signaling in liver cancer cells (Su et al., 2022). E2F is a family of transcription factors that modulate the expression of genes involved in cellular proliferation and cell cycle (Azechi et al., 2001). In kidney cancer, E2F1 could enhance the metastatic potential of tumor cells through the activation of matrix metalloproteinase (MMP) 2 and MMP9 (Ma et al., 2013). Another study had suggested that E2F1 induced the senescence of ccRCC tumor cells *via* p27 (Mans et al., 2013). Therefore, we speculated that CPNE1 might function by E2F to modulate the proliferation ability and cell cycle of ccRCC.

For exploring regulators potentially responsible for CPNE1 dysregulation, we predicted several potential miRNA targets. miR-302c, miR-330, and miR-496 were the mainly miRNA targets of CPNE1 in ccRCC. Several studies have reported that miR-302c is involved in cancer-related processes and positively correlated with the prognosis of cancer. For example, miR-302c was identified as a potent estrogen receptor α-regulatory miRNA and inhibited the estrogen-induced growth in breast cancer (Leivonen et al., 2009; Yoshimoto et al., 2011). Moreover, miR-302c repressed EMT and metastasis *via* targeting TFAP4, and it might serve as a potential prognostic factor and therapeutic target for colorectal cancer (Ma et al., 2018). Increasing miR-330 expression in human colorectal cancer cells was reported to induce apoptosis and suppresses cell viability and migration through inhibition of HMGA2 and Smad3 expression (Mansoori et al., 2020). Elevated miR-330 could overcome cell proliferation, migration, invasion, and metastasis of renal cancer cells (Liu et al., 2022).

Previous studies indicated tumor-infiltrating immune cells in microenvironment serve an essential function in tumor development, metastasis, response to immunotherapy, thus influence prognosis in patients with malignancies (Bindea et al., 2013; Bremnes et al., 2016). Before the initiation of immunotherapy, ccRCC had been known as a typical tumor type to be responsive to immunotherapies, such as cytokine-based regimens (Choueiri and Motzer, 2017). Treatment with immune checkpoint inhibitors have dramatically improved the outcomes of patients with metastatic ccRCC. Thus, exploring new biomarkers or immunotherapeutic targets of ccRCC is of major clinical importance. In the current study, we found that CPNE1 expression was significantly related to immune and estimate scores, but not to stromal score. Interestingly, higher CD8^+^ T cells infiltration level was observed in high CPNE1 expression group, which is related to worse prognosis in KIRC. These results seemed paradoxical. CD8^+^ T cells constitute the majority of microenvironment, which are the major effect cells in anti-tumor immunity (van der Leun et al., 2020). However, the complex and dynamic microenvironment is heterogeneous among different tumor types. In ccRCC, CD8^+^ T cells were related to increased expression levels of immune evasive biomarkers and promoted immune escape. Unlike many other solid tumors, the high infiltration of CD8^+^ T cells in ccRCC predicts poor survival outcomes (Giraldo et al., 2015; Qi et al., 2020). Coherently with the literature, positive relationships were observed between CPNE1 expression and critical immune checkpoint genes, including CTLA4, PDCD1 and LAG3, indicating a tendency for immune evasion. It has been well established that immune checkpoints negatively regulated the immune system (Chen and Mellman, 2017), which once activated would weaken antitumor immune response. Moreover, patients in high CPNE1 expression group exhibited higher TIDE, T-cell exclusion, and T-cell dysfunction scores, which indicated a fewer benefit from immunotherapy and consistent with the results of immune cell infiltration. Therefore, this might be a probable explanation for why high CPNE1 expression group exhibited high CD8+T cell infiltration and had poor prognosis. Overall, these results indicated that CPNE1 might play an important role in regulating tumor immunity, and act as a potential therapeutic biomarker for ccRCC immunotherapy.

In this study, we found CPNE1 might activate the EGFR/STAT3 signaling pathway to promote ccRCC cell growth. Epidermal growth factor receptor (EGFR), a member of tyrosine kinase receptors, was involved in the pathogenesis and progression of many malignancies (Huang et al., 2018). Previous studies have shown that upregulation of EGFR is one of the common events in ccRCC. and it has also been suggested to play a primary role in tumor initiation and progression (Petrides et al., 1990). EGFR phosphorylation activates downstream signaling pathways and increases STAT3 (Lv et al., 2017), AKT (He et al., 2018)and ERK1/2 (Zhou et al., 2020) phosphorylation levels. All these pathways are involved in epithelial-mesenchymal transition (EMT), which is considered to be vital to the cancer metastatic process (Jiang et al., 2019).

However, this study had some limitations. Firstly, our findings were just confirmed in public databases, instead of collecting samples from the real world. Secondly, although we confirmed that CPNE1 could affect EGFR/STAT3 signaling, further intensive studies are needed to elucidate the mechanisms of CPNE1 involved in the development of ccRCC. Lastly, the effect of CPNE1 on tumor immunity will be further investigated.

## 5 Conclusion

In this study, we demonstrated that CPNE1 was significantly upregulated in ccRCC tissues compared to adjacent normal tissues. Elevated CPNE1 expression was related to poor prognosis. Overexpression of CPNE1 promoted the proliferation, migration and invasion through activating the EGFR/STAT3 signaling pathway in ccRCC. In addition, CPNE1 significantly correlated with immune infiltration and immunotherapy responses in ccRCC. In general, our findings suggested that CPNE1 might potentially act as a prognostic biomarker in ccRCC and highlighted the potential value of the CPNE1/EGFR/STAT3 axis as a promising target for combating ccRCC progression.

## Data Availability

The datasets presented in this study can be found in online repositories. The names of the repository/repositories and accession number(s) can be found in the article/Supplementary Material.
