# Supplementary material for: Comprehensive analysis of prognostic value, immune implication and biological function of CPNE1 in clear cell renal cell carcinoma
Source: Front Cell Dev Biol. 2023 Apr 3;11:1157269. doi: 10.3389/fcell.2023.1157269 (PMC10106647; doi:10.3389/fcell.2023.1157269)
Supplement: Supplementary file 2 [file Table1.DOCX]

IDO1

LAG3

CTLA4

TNFRSF9

ICOS

CD80

PDCD1LG2

TIGIT

CD70

TNFSF9

ICOSLG

KIR3DL1

CD86

PDCD1

LAIR1

TNFRSF8

TNFSF15

TNFRSF14

IDO2

CD276

CD40

TNFRSF4

TNFSF14

HHLA2

CD244

CD274

HAVCR2

CD27

BTLA

LGALS9

TMIGD2

CD28

CD48

TNFRSF25

CD40LG

ADORA2A

VTCN1

CD160

CD44

TNFSF18

TNFRSF18

BTNL2

C10orf54

CD200R1

TNFSF4

CD200

NRP1
