# Supplementary figures and images for: Comprehensive analysis of prognostic value, immune implication and biological function of CPNE1 in clear cell renal cell carcinoma
Source: Front Cell Dev Biol. 2023 Apr 3;11:1157269. doi: 10.3389/fcell.2023.1157269 (PMC10106647; doi:10.3389/fcell.2023.1157269)

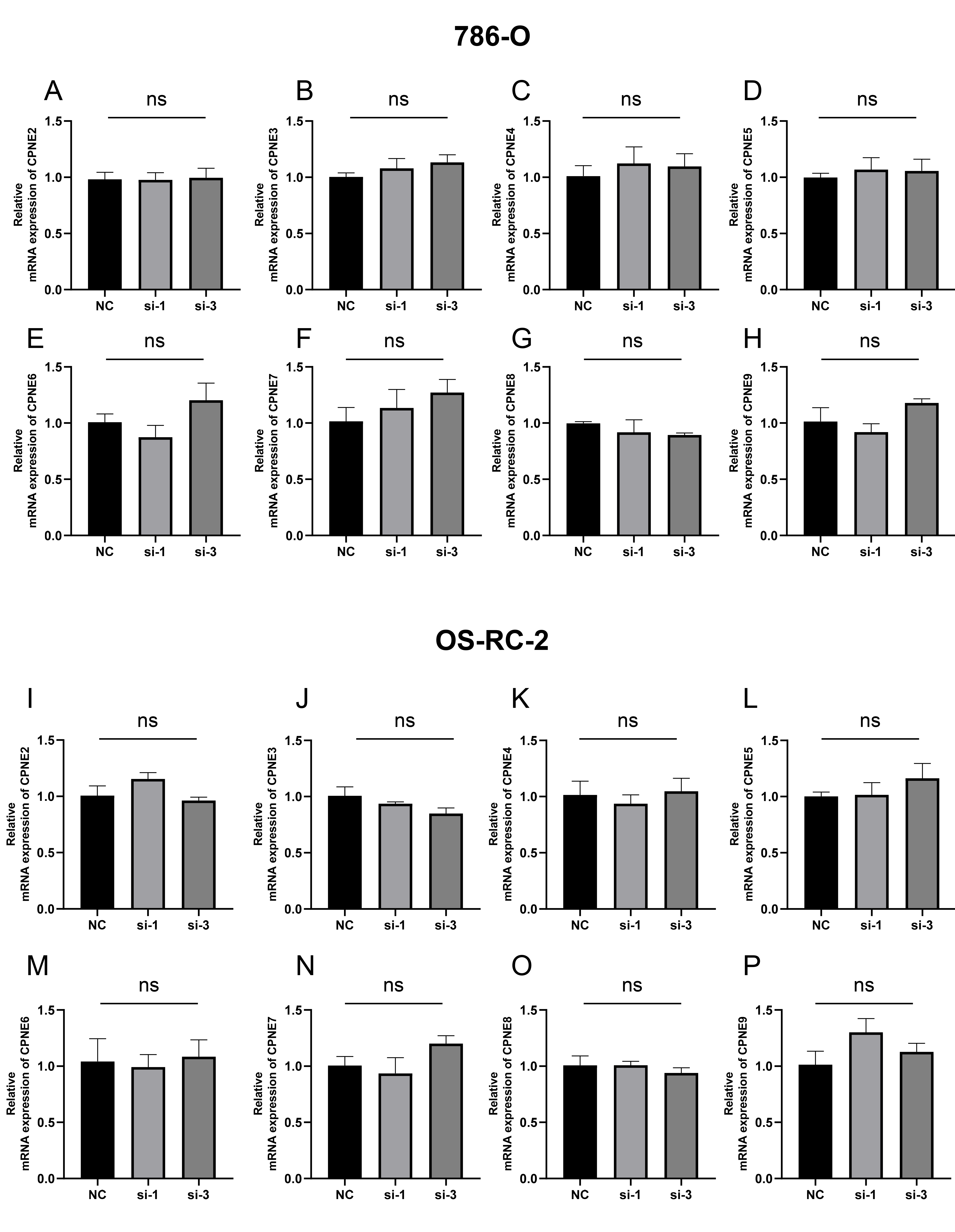

Supplement: Supplementary file 3 [file Image3.TIF]

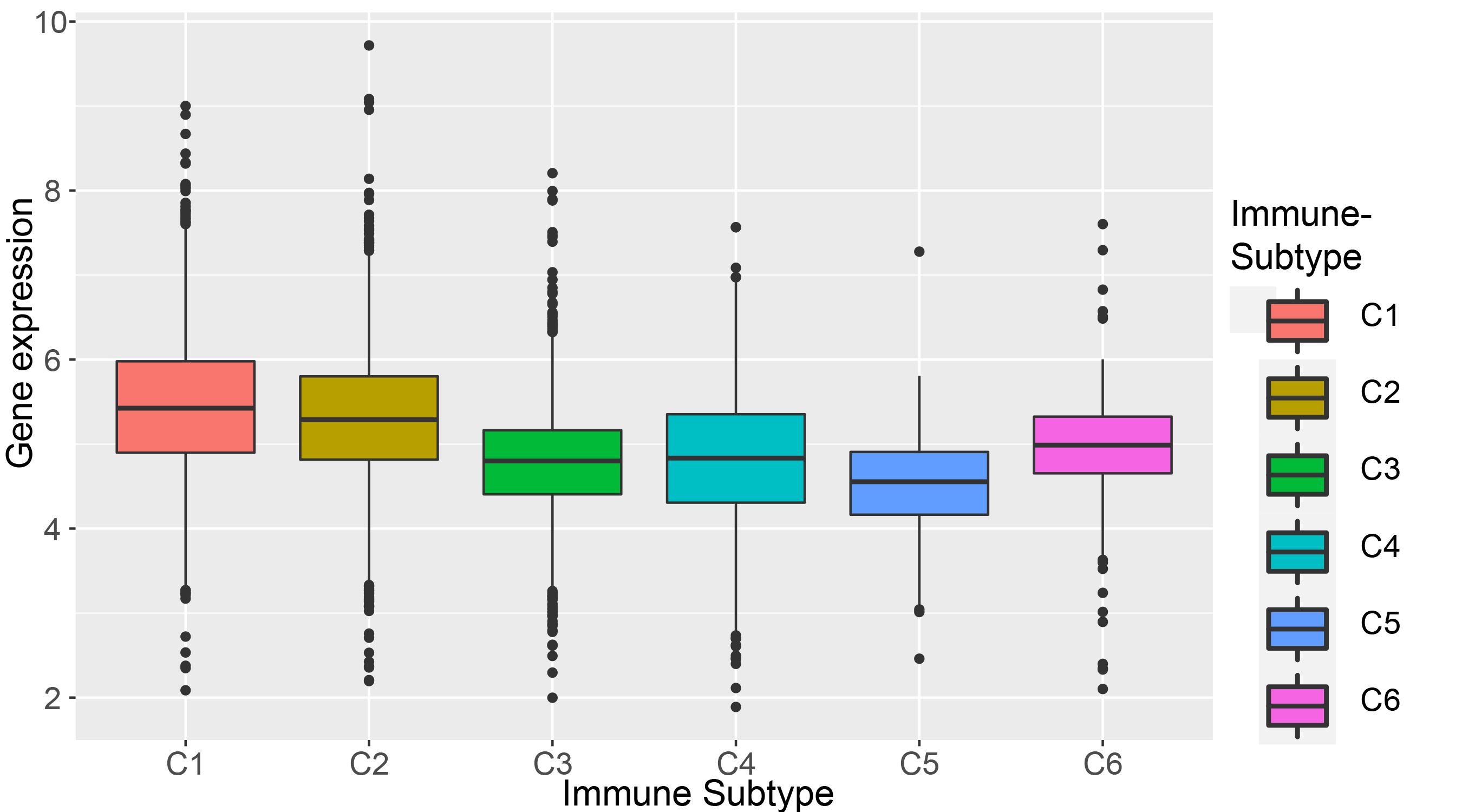

Supplement: Supplementary file 4 [file Image2.TIF]

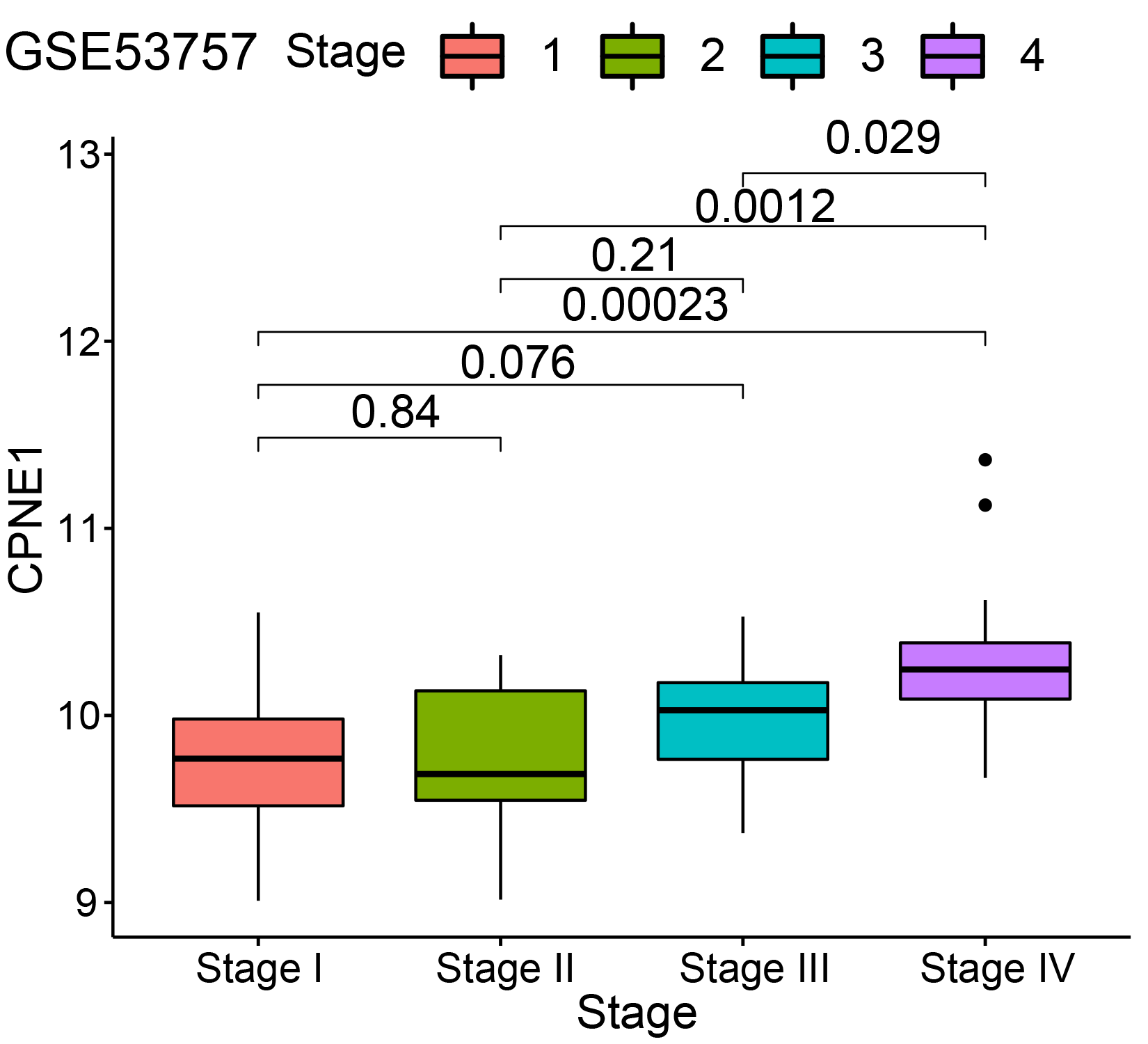

Supplement: Supplementary file 5 [file Image1.TIF]
