# Supplementary material for: Comprehensive analysis of prognostic value, immune implication and biological function of CPNE1 in clear cell renal cell carcinoma
Source: Front Cell Dev Biol. 2023 Apr 3;11:1157269. doi: 10.3389/fcell.2023.1157269 (PMC10106647; doi:10.3389/fcell.2023.1157269)
Supplement: Supplementary file 6 [file Table2.DOCX]

GAPDH

Forward Sequence GTCTCCTCTGACTTCAACAGCG

Reverse Sequence ACCACCCTGTTGCTGTAGCCAA

CPNE1

Forward Sequence CCCTACCAACTTTGCACCCATC

Reverse Sequence ATCCGTCACAGCACCATCAGTC

CPNE2

Forward Sequence GGAAGCCATTCACAGTGCCCTT

Reverse Sequence CTGTGACACTGAGGTCTGGAAC

CPNE3

Forward Sequence GTTTTGGCGCTCAGATACCTCC

Reverse Sequence GACAAGACCGATACGCCTCTAC

CPNE4

Forward Sequence CAGGATACCTCCAGAGTACACG

Reverse Sequence CTCTGATAGGCTTCCACAACTCC

CPNE5

Forward Sequence CACCATGCAGTTCTGTGCCAAC

Reverse Sequence ACCTCGGTCTTGTGGCAAATGG

CPNE6

Forward Sequence TGATACCTTCCTCGGCTCTACG

Reverse Sequence GTCGTTTGTGCCTGATACCTCC

CPNE7

Forward Sequence AACGCTGGCAAGTCCACCATCA

Reverse Sequence TGAAGAGGTCCTTGTCGTCCAG

CPNE8

Forward Sequence GCAAAACTGCCTCCAGATGGAAG

Reverse Sequence TCAGACTCCTGTAATAAGCCTCC

CPNE9

Forward Sequence GCTCCTGTCATCAACCAAGTGG

Reverse Sequence CTCCTTGGTCTGCGTCATGTCA
